# Veno-arterial extracorporeal membrane oxygenation for respiratory and cardiac support in neonates: a single center experience

**DOI:** 10.3389/fcvm.2024.1322231

**Published:** 2024-02-07

**Authors:** Gang Wang, Qiuping Li, Gengxu Zhou, Xiaoyang Hong, Zhe Zhao, Qiang Meng, Zhichun Feng

**Affiliations:** ^1^ The Second School of Clinical Medicine, Southern Medical University, Guangzhou, China; ^2^ Department of Pediatric Cardiac Surgery, The Seventh Medical Center of the PLA General Hospital, Beijing, China; ^3^ Department of Pediatrics, The Seventh Medical Center of the PLA General Hospital, Beijing, China

**Keywords:** extracorporeal membrane oxygenation, neonates, cannulation, respiratory support, cardiac support

## Abstract

**Objective:**

Extracorporeal membrane oxygenation (ECMO) is an advanced life support that has been utilized in the neonate for refractory respiratory and circulatory failure. Striving for the best outcomes and understanding optimal surgical techniques continue to be at the forefront of discussion and research. This study presents a single-center experience of cervically cannulated neonatal patients on V-A ECMO, a description of our cannulation/decannulation techniques and our patient outcomes.

**Methods:**

Single center retrospective review of neonates who received neck V-A ECMO support from January 2012 to December 2022. The data and outcomes of the patients were retrospectively analyzed.

**Results:**

A total of 78 neonates received V-A ECMO support. There were 66 patients that received ECMO for respiratory support, the other 12 patients that received ECMO for cardiac support. The median duration of ECMO support was 109 (32–293) hours for all patients. During ECMO support, 20 patients died and 5 patients discontinued treatment due to poor outcome or the cost. A total of 53 (68%) patients were successfully weaned from ECMO, but 3 of them died in the subsequent treatment. Overall 50 (64%) patients survived to hospital discharge. In this study, 48 patients were cannulated using the vessel sparing technique, the other 30 patients were cannulated using the ligation technique. We found no significant difference in the rates of normal cranial MRI at discharge between survivors with and without common carotid artery ligation.

**Conclusion:**

We achieved satisfactory outcomes of neonatal ECMO in 11-year experience. This study found no significant difference in early neuroimaging between survivors with and without common carotid artery ligation. The long-term neurological function of ECMO survivors warranted further follow-up and study.

## Introduction

Extracorporeal membrane oxygenation (ECMO) is an advanced life support for severe cardiovascular and/or respiratory failure that is unresponsive to conventional therapies. ECMO has been used in newborns for almost 50 years, since Dr. Robert Bartlett successfully used ECMO to support a neonate with hypoxic respiratory failure from meconium aspiration in 1975 (1). From 1989 to 2022, the Extracorporeal Life Support Organization (ELSO) registry reported 48,373 neonates supported with ECMO worldwide (2). In the last decade, while pulmonary disease remained the primary diagnosis for neonatal ECMO, the number of neonatal cardiac ECMO cases had gradually increased (2). Currently, V-A ECMO is still the predominant neonatal ECMO mode. Cannulation via right cervical vessels is a standard method for establishing V-A ECMO support in neonates. Neonatal ECMO cannulation remains a challenging procedure, due to delicate vessels and critical condition. Although there have been many reports of surgical technique for neck V-A ECMO cannulation and decannulation in neonates (3–5), there is still no consensus on the carotid artery ligation or repair during decannulation. Our hospital is a tertiary referral center in Mainland China and started neonatal and children ECMO from 2008. In this study, we retrospectively reviewed the outcomes of V-A ECMO support in neonates at a single center and described our cannulation and decannulation techniques.

## Materials and methods

This was a retrospective review of all neonates (age ≤ 28 days) who received V-A ECMO support in the Department of Pediatric Intensive Care Unit, The Seventh Medical Center of the PLA General Hospital from January 2012 to December 2022. This study was approved by the Institutional Review Board of The Seventh Medical Center of the PLA General Hospital. Patients' data were retrospectively collected from the hospital medical records, including demographic characteristics, etiology for ECMO, surgical technique, duration of ECMO support, survival to hospital discharge and major complications.

### Inclusion criteria

Patients were considered for ECMO support if they met the following criteria: (1) Oxygenation index (OI) > 40 for >4 h or OI > 20 for >24 h; (2) Severe hypoxic respiratory failure with acute decompensation (PaO2 < 40 mmHg) that failed to respond to maximal medical therapy, including high frequency ventilation, use of inhaled nitric oxide; (3) Severe pulmonary hypertension with evidence of right ventricular dysfunction and/or left ventricular dysfunction; (4) shock and refractory hypotension with evidence end-organ malperfusion despite maximal medical therapy. Patients with congenital heart disease who received ECMO for preoperative stabilization and postoperative support were excluded from this study.

### ECMO management

All patients in this study received VA mode ECMO support, cannulations were performed via the right common carotid artery and internal jugular vein. The centrifugal pump was used in all patients. We generally maintained a pump flow rate of 80–120 ml/kg/min in order to achieve adequate tissue perfusion and gas exchange, the target mixed venous oxygenation saturation was 70%–80%. Once the patient was stabilized on ECMO support, the ventilator was adjusted to the “rest mode”, as peak inspiratory pressure (PIP) 15–22 cmH_2_O, positive end-expiratory pressure (PEEP) 5–8 cmH_2_O, rate 10–20/min, oxygen fraction 0.21–0.3. Continuous heparin infusion was used for anticoagulation, and activated clotting time (ACT) was maintained around 180–220 s.

We assessed the cardiopulmonary function of the patients daily by bedside ultrasound and chest x-ray. Once the heart contractility improved and lung function recovered, a trial-off support was performed. When pump flow rate was decreased to less than 50 ml/kg/min, the patients were ready for decannulation if hemodynamics and oxygenation were stable.

### Cannulation techniques

The patient was in the supine position. An shoulder roll was placed to extend the neck up and to the left. A transverse incision about 1.5–2 cm long was made at the right lesser supraclavicular fossa. We prefered to make the incision along the langer line of the neck to achieve aesthetic effect. The platysma was dissected and identified the sternocleidomastoid muscle. Subsequently, the sternal head and the clavicular head of the sternocleidomastoid muscle were separated laterally to expose the carotid sheath. The carotid sheath was opened, then the common carotid artery and the internal jugular vein were very carefully dissected to allow space for cannulation and clamp, taking care not to cause vasospasm and damage the vagus nerve.

### Arterial cannulation

We were used to insert the arterial cannula firstly. There were two techniques for artery cannulation, ligation technique and vessel sparing technique.

Ligation technique: We placed a vessel loop around the proximal common carotid artery, ligated the distal artery with 4^#^ silk suture. The proximal artery was clamped, and the artery was opened using an 11^#^ blade scalpel, the opening was gently dilated with a mosquito clamp. The cannula was inserted and the proximal artery clamp was opened. The cannula was secured using vessel loop and tied with the cannula using 10^#^ silk suture.

Vessel sparing technique: We placed a purse string suture using 5-0 polypropylene on the anterior wall of the carotid artery. The proximal and distal artery were clamped, the cannula was inserted through the center of the purse string and the proximal artery clamp was opened. The cannula was secured with a Rummel tourniquet which were tied to the cannula using 10^#^ silk suture, and then the distal artery clamp was opened.

We chose 8−10 F arterial cannula, and the insertion depth of the carotid artery cannula was usually 2 cm in neonates.

### Venous cannulation

In neonates, we performed internal jugular vein cannulation by the ligation technique described above, chose 8–12 F venous cannula. Prior to cannulation, the insertion depth of the venous cannula should be estimated from the venotomy to the level of the line between the two nipples. Generally, the depth of the internal jugular vein cannula was about 6–7 cm.

Once the cannulations were completed, the ECMO support should be initiated. We observed whether the pump flow was satisfactory and confirmed the position of the cannulas by bedside echocardiogram. Finally, the skin incision was closed with interrupted silk sutures, and then the cannulas were secured to the skin with sutures.

### Decannulation techniques

For decannulation, preoperative preparation and position were the same as for cannulation. The neck incision was reopened, and the common carotid artery and internal jugular vein were exposed. If the distal artery was already ligated during cannulation, the proximal artery was ligated with a silk suture when the cannula was removed. If vessel sparing technique was used during cannulation, the tourniquet was removed and the purse-string suture was tied down as the cannula was removed. If there was bleeding at the cannulation site, we would reinforce with sutures of 5-0 polypropylene. When the venous cannula was removed, we routinely ligated the proximal vein.

### Statistical analysis

Data were analyzed with SPSS 25 software. Continuous variables were analyzed by using the Mann–Whitney *U* test. Categorical variables were compared using the Chi-square test, and if the expected number of observations was <5, Fisher's exact test was used. *p* values of less than 0.05 were considered statistical significant.

## Results

During the study period, a total of 78 neonates received V-A ECMO support. The patients' characteristics and clinical data were shown in Table 1. There were 53 male (68%) and 25 female (32%). Of the 78 patients, 5 patients were placed on ECMO by our ECMO team in other hospitals and then transported to our institution. The median gestational age (GA) was 39 weeks (range, 37–40 weeks), and the median birth weight was 3.2 kg (range, 3.0–3.6 kg). The median age received ECMO was 2 days (range, 1–5 days).

**Table 1:** Characteristics of neonates who received V-A ECMO support.

Characteristics (*n* = 78)	Data *n* (%) or median (range)
Gestational age (weeks)	39 (37–40)
Birth body weight (kg)	3.2 (3.0–3.6)
Sex (male/female)	53/25
Apgar score at 1 min	5.5 (3–8)
Apgar score at 5 min	6 (4.75–9.25)
Age received ECMO (days)	2 (1–5)
Etiology	
MAS	25 (32.1)
PPHN	17 (21.8)
RDS	13 (16.7)
Sepsis	5 (6.4)
CDH	3 (3.8)
Pneumonia	2 (2.6)
Pulmonary dysplasia	1 (1.3)
Septic shock	6 (7.7)
Cardiac arrest	3 (3.8)
Myocarditis	2 (2.6)
Congenital atrioventricular block	1 (1.3)
Duration of ECMO support(hours)	109 (32–293)
Survive to hospital discharge	50 (64)

ECMO, extracorporeal membrane oxygenation; MAS, meconium aspiration syndrome; PPHN, persistent pulmonary hypertension of the newborn; RDS, respiratory distress syndrome; CDH, congenital diaphragmatic hernia.

There were 66 patients that received ECMO for respiratory support, etiology included meconium aspiration syndrome (MAS, *n* = 25), persistent pulmonary hypertension of the newborn (PPHN, *n* = 17), respiratory distress syndrome (RDS, *n* = 13), sepsis (*n* = 5), congenital diaphragmatic hernia (CDH, *n* = 3), pneumonia (*n* = 2), pulmonary dysplasia (*n* = 1). The other 12 patients that received ECMO for cardiac support, etiology included septic shock (*n* = 6), cardiac arrest (*n* = 3), myocarditis (*n* = 2), congenital atrioventricular block (*n* = 1).

The median duration of ECMO support was 109 (32–293) h for all patients. During ECMO support, 20 patients died (renal failure in 7, multiple organs failure in 5, heart failure in 3, parenchymal hemorrhage in 3, severe infection in 2), 3 patients discontinued treatment due to poor outcome, and 2 patients' families withdrawed treatment because of the cost. A total of 53 (68%) patients were successfully weaned from ECMO, but 3 of them died in the subsequent treatment, the causes of death were cerebral hemorrhage, renal failure, and infection, respectively. Overall 50 (64%) patients survived to hospital discharge. According to the etiology, the rate of survival to hospital discharge was as follows: MAS,72% (18/25); PPHN, 65% (11/17); RDS, 69% (9/13); sepsis, 40% (2/5); CDH, 33% (1/3); pneumonia, 100% (2/2); pulmonary dysplasia, 0% (0/1); septic shock, 50% (3/6); cardiac arrest, 67% (2/3); myocarditis, 50% (1/2); congenital atrioventricular block, 100% (1/1). There was no statistically significant difference in the rate of survival to hospital discharge between respiratory support ECMO and cardiac support ECMO (65% vs. 58%, *p *= 0.900; Table 2).

**Table 2:** Etiology and survival to discharge rate.

Etiology	Survival to discharge rate (%)
Respiratory support	65*
MAS	72
PPHN	65
RDS	69
Sepsis	40
CDH	33
Pneumonia	100
Pulmonary dysplasia	0
Cardiac support	58*
Septic shock	50
Cardiac arrest	67
Myocarditis	50
Congenital atrioventricular block	100

MAS, meconium aspiration syndrome; PPHN, persistent pulmonary hypertension of the newborn; RDS, respiratory distress syndrome; CDH, congenital diaphragmatic hernia.

* There was no statistically significant difference in the rate of survival to hospital discharge between respiratory support ECMO and cardiac support ECMO (*p *= 0.900).

**Figure 1:**
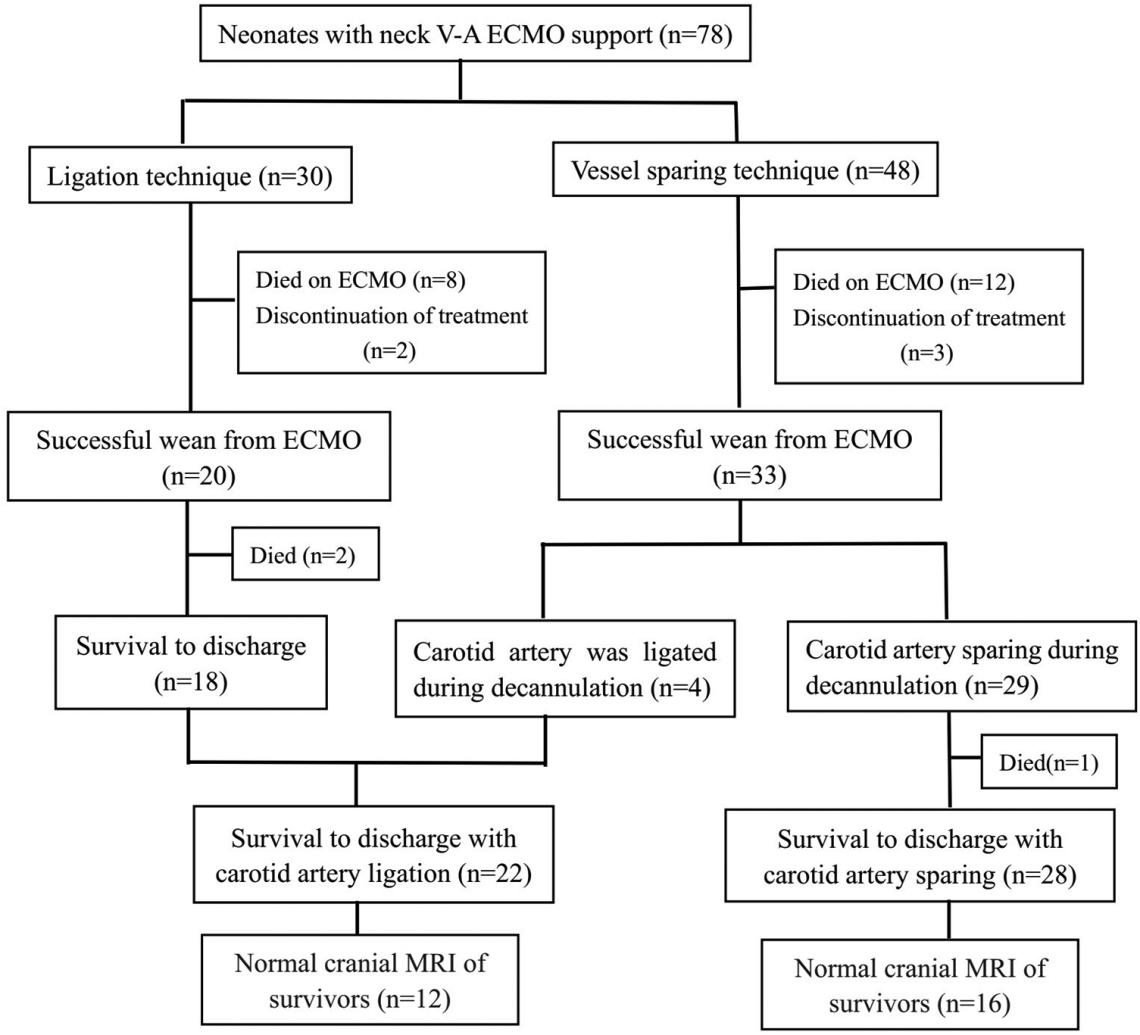
Cannulation technique and outcomes of neonatal V-A ECMO.

In our hospital, the surgeon determined the cannulation strategy of neonatal ECMO. In this study, 48 patients were cannulated using the vessel sparing technique, of which 33 patients were successfully weaned from ECMO. Furthermore, 4 of the 33 patients underwent carotid artery ligation at the time of decannulation due to poor vessel condition. The other 30 patients were cannulated using the ligation technique (Figure 1). The neck vessels were evaluated by ultrasound before discharge in patients without carotid artery ligation, and the common carotid artery patency rate was 100%.

The major complictions were as follows, circuit clotting was diagnosed in 10 patients, acute kidney injury in 8 patients, cerebral hemorrhage in 5, cerebral infarction in 3, cannulation site bleeding in 3, pericardial effusion in 1, hemolysis in 3, culture positive infection in 4.

To determine whether there was central nervous damage, all survivors would receive a cranial magnetic resonance imaging (MRI) before discharge. Among the survivors of carotid ligation, there were focal infarction in 2 patients, ventricular dilatation in 4, wide interhemispheric fissure in 2, cerebral atrophy in 2, and normal in 12 patients (54.5%). Among the survivors of vessel sparing, there were focal infarction in 1 patients, intraventricular hemorrhage in 1, ventricular dilatation in 5, wide interhemispheric fissure in 2, large subarachnoid space in 1, cerebral atrophy in 2, and normal in 16 patients (57.1%). We found no significant difference in the rates of normal cranial MRI at discharge between patients with and without common carotid artery ligation (54.5% vs. 57.1%, *p *= 0.854; Table 3).

**Table 3:** Characteristics of ECMO survivors.

	Ligation (*n* = 22)	Vessel sparing (*n* = 28)	*p*-value
Gestational age (weeks)	39 (37.75–39.25)	39 (38–40)	0.595
Birth body weight (kg)	3.30 (3.07–3.80)	3.20 (3.10–3.55)	0.403
Apgar score at 1 min	5 (3–8)	5.5 (3.25–7)	0.922
Apgar score at 5 min	6 (4.75–8.25)	6 (4–8)	0.594
Age received ECMO (days)	2 (1–4.25)	2 (1–3.75)	0.835
Normal cranial MRI of survivors, *n* (%)	12 (54.5)	16 (57.1)	0.854

## Discussion

In the present study, we reviewed a single center experience with neonatal V-A ECMO in Mainland China. The development of ECMO for children and newborns in Mainland China was relatively late. Our hospital started this technology in 2008 and was one of the earliest institutions in Mainland China to carry out ECMO for neonatal patients. In this study, all patients received ECMO support according to the ELSO guideline. A total of 78 neonates received V-A ECMO support, and 50 (64%) patients survived to hospital discharge. Overall survival to discharge rate was similar to those in the ELSO Registry report (64% vs. 65%) (2). In this study, we found no statistically significant difference in the rate of survival to hospital discharge between respiratory support ECMO and cardiac support ECMO (65% vs. 58%, *p *= 0.900). However, compared to data from the ELSO registry report, the survival to discharge rate of respiratory support ECMO was lower, and the survival to discharge rate of cardiac support ECMO was higher in the present study. We believe that due to the high mortality rate of neonates treated with ECMO for CDH in our center, the survival to discharge rate of respiratory support ECMO in the present study is lower than that of ELSO Registry (65% vs.72%) (2). ECMO support for neonatal CDH had the worst outcome among all the neonatal respiratory failure diseases. ECMO for CDH has a survival rate of around 50% that has remained unchanged over time (2, 6–8). However, the survival to discharge rate of ECMO for CDH was only 33% in our center. Because we did not include patients with congenital heart disease, the survival to discharge rate of cardiac support ECMO was higher than that reported by ELSO Registry (58% vs. 44%) (2).

In our report, MAS, PPHN, and neonatal respiratory distress syndrome were the most common indications for ECMO, accounting for 71% of the total. CDH in 3 cases, only 3.8% of the total. Twelve patients received ECMO for cardiac support, accounting for 15% of the total. From the above data, it can be found that our hospital is different from data of ELSO registry in terms of etiology of ECMO (2). Nowadays, CDH has become the most common ECMO indication in the neonatal period (2). But, the number of neonates with CDH treated by ECMO in our center was very low. We speculated that the reasons may be as follows: on the one hand, some fetuses were aborted after the prenatal diagnosis of CDH, on the other hand, the parents of the patients gave up treatment due to poor prognosis.

According to data from the Extracorporeal Life Support Organization (ELSO) registry, the number of ECMO for neonatal cardiac support had increased in the last decade (2). The congenital heart defects were the most frequent indication of ECMO for cardiac support (2, 9, 10). In this study, we excluded the patients with congenital heart disease who received ECMO, because postoperative patients underwent ECMO cannulation through an open sternotomy, not through the cervical vessels. Therefore, the proportion of ECMO for cardiac support was lower in our study than that reported by ELSO Registry (15% vs. 22%) (2).

Because the double-lumen cannula had not been registered in the Mainland China, all neonatal ECMO were V-A mode in our center. Peripheral ECMO in neonates and infants were established through the right cervical vessels, cannulation and decannulation were performed by pediatric cardiac surgeon in our hospital. For infants and children, both the common carotid artery and the internal jugular vein cannulation were performed using vessel sparing techniques, and the vessels were repaired during decannulation. However, in neonates, the internal jugular vein was ligated during cannulation, the common carotid artery was cannulated using ligation or vessel sparing technique was at the discretion of the surgeon. But, at the time of decannulation, if the carotid artery damage was severe, we would ligate the artery even if the vessel sparing technique was used during cannulation. We considered that there is a high risk of anastomotic bleeding, aneurysm, and stenosis when repairing severely damaged arteries. This vessel sparing technique we described is to secured cannula with purse string and Rummel tourniquet. This is a simple technique, with less damage to vessel. In this report, the patency rate of the common carotid artery was 100% at discharge with vessel sparing technique. Studies on long-term patency of carotid artery had reported inconsistent results. Buesing et al. (11) reported that more than half of the reconstructed common caroid arteries were either occlusive or severely stenotic in a long-term MRI follow-up study. However, Duggan et al. (4) reported that 31 of 37 (84%) neonates who underwent vessel repair had patent carotid arteries at a median of 63 days (range 0–4,231 days) after decannulation. In another study, all 11 infants who were placed on ECMO using vessel sparing technique had patency of the artery at a median of 127 days (range 37–400 days) after decannulation (12). In the present study, we achieved excellent early patency rate of caroid artery with vessel sparing technique, the long-term outcomes of vessel patency needed further follow-up.

Cannulation via right cervical vessels is the most common method for establishing neonatal V-A ECMO. However, there is still no consensus on the vessels management during decannulation. The main controversial issue is carotid artery ligation or repair after ECMO. On the one hand, ligation of the right carotid artery possibly lead to right hemisphere injury (13, 14); on the other hand, carotid repair has the potential risks of stroke, anastomotic bleeding, and aneurysm (15, 16). Intraventricular hemorrhage, periventricular leukomalacia, ischemia, cerebellar or cortical hemorrhage, infarct, hydrocephalus, and diffuse atrophy were typical abnormal imaging findings in neonates and children who underwent ECMO support (17–19). Lago et al. (20) found that asymmetric cerebrovascular after right carotid artery ligation may lead to local cerebral lesions by magnetic resonance angiography. Other studies had showed abnormal imaging findings after ligation as well (21, 22). Conversely, Campbell et al. (13) did not observe any increase in right-sided brain lesions on imaging studies of the infants who underwent carotid artery ligation. McCutcheon et al. (23) also found no difference between the two cerebral hemispheres in children with the right common carotid artery ligation. In this study, We evaluated neurologic lesions by cranial MRI, and found no significant difference in the rates of normal cranial MRI at discharge between patients with and without common carotid artery ligation (54.5% vs. 57.1%, *p *= 0.854). We speculated that these mixed findings may be related to underlying severe condition of the patients and the congenital anatomic variations of the circle of Willis. Some studies had showed that compensation of the circle of Willis was very important in neonates with carotid artery ligation (24, 25). In the present study, we did not evaluate the circle of Willis in all patients, and futher studies need to include this aspect.

At present, there is no definite evidence that either reconstruction or ligation will lead to long-term neurological abnormalities. Some studies on the long-term neurological sequela in neonates who underwent ECMO had found that there was little difference in imaging, intelligence quotient, developmental scores or rate of cerebral palsy between carotid artery ligation group and reconstruction group (22, 25–27). In this study, we found no significant difference in early neuroimaging between survivors with and without common carotid artery ligation. The long-term neurological function need to further follow-up and study.

We acknowledge that this study has some limitations. This is a single center retrospective study with a small sample size and heterogeneous patient population. In addition, the long-term carotid artery patency rate and the long-term neurological function of the study cohort had not been included.

## Conclusion

Although our center started undertaking neonatal ECMO later than other major centers, our study showed satisfactory outcomes in our 11-year experience. The vessel sparing technique we described is simple and practical. This study demonstrated excellent early patency rate of the common carotid artery with vessel sparing technique. We also found that there was no significant difference in early neuroimaging between survivors with and without common carotid artery ligation. The long-term outcomes of carotid artery patency and neurological function need to further follow-up and study.

## Data Availability

The original contributions presented in the study are included in the article/Supplementary Material, further inquiries can be directed to the corresponding author.
